# EGFRvIII TandAbs are specific and highly potent drug candidates for the treatment of solid tumors

**DOI:** 10.1186/2051-1426-3-S2-P219

**Published:** 2015-11-04

**Authors:** Kristina Ellwanger, Uwe Reusch, Ivica Fucek, Michael Weichel, Thorsten Gantke, Stefan Knackmuss, Vera Molkenthin, Jens-Peter Marschner, Martin Treder

**Affiliations:** 1 Affimed GmbH, Heidelberg, Germany; 2 AbCheck s.r.o., Plzen, Czech Republic

## 

To harness the immune system's cytotoxic capacity to fight solid tumors, we developed tetravalent, bifunctional antibodies that recognize EGFRvIII, the deletion variant III of EGFR, and either CD3 or CD16A on immune cells, thereby directing T cells or NK-cells to eliminate EGFRvIII^+^ cancer cells.

Using phage display, we identified scFv antibodies that selectively bind to EGFRvIII. These highly EGFRvIII-specific scFv antibodies were substantially improved by affinity maturation achieving K_D_s in the 100 pM range or lower and used to construct a set of bispecific EGFRvIII-targeting TandAbs with a broad range of binding and cytotoxic properties. Mono- and bivalent binding constants, specificity for EGFRvIII and CD3 or CD16A, cytotoxic activity, and target-dependent effector cell activation were characterized in a panel of *in vitro* assays. TandAbs exhibited exquisite specificity towards the EGFRvIII antigen in Western Blot, SPR, ELISA, and FACS assays of EGFRvIII^+^ cells. No binding was observed to recombinant EGFR or to EGFR^+^ cells. The TandAbs apparent affinities for EGFRvIII were up to 25-fold improved relative to the monovalently binding scFvs, resulting in a K_D_ of 11 pM for the best TandAb.

EGFRvIII/CD3 and EGFRvIII/CD16A TandAbs with high affinity for EGFRvIII were similarly potent in killing assays, displaying cytotoxicity towards EGFRvIII^+^ F98 glioma, transfected CHO or human DKMG cells with EC_50_ in the range of 1 pM – 10 pM. No cytotoxicity was observed on EGFR^+^ cells or EGFRvIII-negative cells demonstrating the high selectivity of EGFRvIII TandAbs for the tumor-specific EGFRvIII. Importantly, in the absence of EGFRvIII^+^ target cells *in vitro* TandAbs did not elicit T- or NK-cell activation, as demonstrated by their lack of proliferation. Binding to EGFRvIII in different solid tumor types and its absence from healthy tissues was shown by immunohistochemistry using a high affinity EGFRvIII-binding bivalent Diabody.

In summary, EGFRvIII/CD3 and EGFRvIII/CD16A TandAbs provide an opportunity to develop cytotoxic antibodies that solely target cancer, sparing normal tissues and thereby reduce the side effects associated with EGFR therapy.

